# Peripapillary and Macular Flow Changes in Nonarteritic Anterior Ischemic Optic Neuropathy (NAION) by Optical Coherence Tomography Angiography (OCT-A)

**DOI:** 10.1155/2020/3010631

**Published:** 2020-11-02

**Authors:** Juejun Liu, Changzheng Chen, Lu Li, Zuohuizi Yi, Hongmei Zheng

**Affiliations:** Eye Center, Renmin Hospital of Wuhan University, Wuhan, China

## Abstract

**Background:**

To analyze the blood flow changes of radial peripapillary capillaries (RPCs) and macula with time procession in patients with nonarteritic anterior ischemic optic neuropathy (NAION) by optical coherence tomography angiography (OCT-A).

**Methods:**

A total of 21 affected eyes and 19 unaffected eyes from 21 NAION patients were included. Assessments of BCVA, CFP, SD-OCT, and OCT-A were performed on NAION patients at enrollment and at 1-2 weeks, 1-2 months, and 3–6 months after enrollment. Measures of the thickness of the peripapillary retinal nerve fiber layer (wRNFL) and macular ganglion cell complex (wGCC) of the whole image in SD-OCT, vessel density of the RPC (wRPC) and superficial and deep vascular complexes (wSVD, wDVD) in the whole image of OCT-A, and their superior- and inferior-hemi values (s/iRNFL, s/iGCC, s/iRPC, and s/iSVD) were assessed.

**Results:**

Compared to unaffected control eyes, wRPC (*p* ≤ 0.001) was significantly lower in affected eyes at baseline, and there was no significant difference in wSVD (*p* > 0.05). The wRPC and wSVD values of affected eyes were significantly decreased at follow-up time points of 1–2 and 3–6 months compared to baseline (*p*=0.001, *p* ≤ 0.001; *p* ≤ 0.001, *p* ≤ 0.001). The sRPC values were significantly lower than iRPC at 1-2/3–6 months (*p*=0.016, *p*=0.013), and sSVD values were lower than iSVD at 1-2 months (*p*=0.010). Statistically significant correlations were found between wRPC and wRNFL values at 3–6 months (*r* = 0.626, *p*=0.022), between wSVD and wGCC at 1-2 weeks and 1-2 months (*r* = 0.570, *r* = 0.436; *p*=0.007, *p*=0.048).

**Conclusion:**

OCT-A revealed a sectorial reduction in vessel density in the RPC and macula with the disease progression of NAION from acute to atrophic stages, a classification associated with structural deficits.

## 1. Introduction

Nonarteritic ischemic optic neuropathy (NAION) is the most common subtype of ischemic optic neuropathy occurring in middle-aged and elderly patients [1, 2], which might be related to transient hypoperfusion or nonperfusion of the optic nerve head (ONH) [1]. Optical coherence tomography (OCT) has been previously used to evaluate the initial state of optic disc edema (ODE) in the course of NAION and monitor the optic nerve injury and subsequent atrophy [3, 4]. Compared with traditional fundus angiography and Doppler imaging that are used to confirm a reduction in flow to the ONH [1, 5, 6], OCT-A makes it possible to noninvasively observe and quantify the microvasculature in the optic disc and the macular retina over the selected layers [7–10].

Research regarding the applications of OCT-A in NAION has attracted attention over the last several years; however, most of the resulting work produced small cross-sectional studies with a limited sample size focusing on postacute NAION with resolved ODE [9–12]. A few studies were designed to follow-up the capillary density changes of the peripapillary radial capillaries and the capillary density in the layers of the macula from acute to atrophic stages [13]. Measurements in previous cross-sectional studies are likely not interchangeable given that the results are from different stages of NAION with different classification criteria and blood flow parameters in variant measuring methods. This is the first study to follow-up a group of patients with NAION by OCT-A. This novel technique is used here to quantitatively document the microvascular flow impairments in the peripapillary capillary of ONH and the macular retina over time and to evaluate the corresponding structural deficits.

## 2. Materials and Methods

### 2.1. Retrospective Cohort Study

Patients with NAION were enrolled within 2 weeks of onset at the Eye Center of the Renmin Hospital of Wuhan University between November 2017 and June 2019. All patients received thorough ophthalmic examinations at the initial visit, including best-corrected visual acuity (BCVA) and visual field assessments (Humphrey perimetry, Zeiss, Germany), Fundus fluorescein angiography (FFA) (HRA2; Heidelberg Engineering, Heidelberg, Germany), color fundus photography (CFP) (Zeiss, Oberkochen, Germany; Optomap 200Tx, Optos, Marlborough, MA, USA), spectral-domain OCT (SD-OCT), and OCT-A (RTVue XR AngioVue Version 2017.1; Optovue Inc. Fremont, CA, USA) analyses. The inclusion criteria for patients with NAION are defined as follows [1]: a complain of sudden and unilateral painless loss in visual acuity and/or visual field defect within 2 weeks; optic disc edema (ODE) associated with or without peripapillary hemorrhages at the initial visit, and the resolution of ODE during follow-up has been observed; occurrence of visual field impairment with a relative altitudinal pattern (which are usually nasal and/or inferior); and segmental filling defect/delay in the optic disc during the acute stage of fluorescein fundus angiography (FFA). All patients had normal C-reactive protein blood test results, and CT and/or MRI examinations of the skull and orbit were used to exclude A-AION and other nervous system diseases affecting visual function and the visual field or causing optic disc edema. Patients with evidence of any other condition that could damage the optic disc (e.g., glaucoma, uveitis, optic neuritis, or diabetic retinopathy) and a history of ocular surgery were excluded. All patients were administered systemic corticosteroid therapy with oral prednisone 80 mg for the first 2 weeks, 70 mg for 1 week, 60 mg for 1 week, and then, gradually decreased [1, 14]. The diagnosis was confirmed by one of the investigators who are professional experts. All subjects who had myopia more than 6.0 diopters and severe cataracts that could impair the scan quality of OCT-A were excluded from this cohort. Quantitative and noninvasive assessments of BCVA, CFP, SD-OCT, and OCT-A were performed at the initial visit and repeated in patients of NAION at 1–2 weeks, 1–2 months, and 3–6 months after enrollment. According to the literature [1, 2, 4], the acute phase of NAION in this study was determined at the initial visit at our clinic, which was within 2 weeks of noticing visual symptoms. The follow-up intervals 1–2 weeks and 1–2 months counting from the time of enrollment generally correspond to the stage of subacute and resolution in NAION, and 3–6 months corresponds to the chronic atrophic stage.

SD-OCT was performed in two modes including Nerve Fiber-ONH within a 4.9 mm diameter circle centered on the optic disc and Nerve Fiber-GCC within a 6 × 6 mm scan area centered 1 mm temporal to the fovea. Automatic quantitative parameters were recorded as follows: average thickness of the peripapillary retinal nerve fiber layer (wRNFL, *μ*m) and macular ganglion cell complex of the whole image (wGCC, *μ*m; encompasses three layers of retinal nerve fiber layer (RNFL), ganglion cell layer (GCL) and inner-plexiform layer (IPL)), and their superior and inferior values (sRNFL, *μ*m, iRNFL, *μ*m; sGCC, *μ*m, iGCC, *μ*m).

Capillary density of the radial peripapillary capillary (RPC) was measured within a 4.5 × 4.5 mm scan area centered on the optic disk in the mode of Angio-Disc 4.5 mm, and vessel density of macular retina was measured within a 3 × 3 mm scan area centered on the fovea in the Angio-Retina 3.0 mm mode. Angio-OCT scanning was acquired using the eye tracking option. Multiple scans (more than twice) were performed, and images with a quality score greater than or equal to 7/10 and without eye movement artifacts were preserved. Automatic quantitative parameters of the optic disc include average capillary density of RPC based on the whole image (wRPC, %) and the superior- and inferior-hemi values (sRPC, %; iRPC, %) based on the Garway-Heath peripapillary grid (the peripapillary region is defined by two rings of 2 mm and 4 mm centered on the disc center using deep Bruch's membrane opening as a reference for the disc margins). Segmentation of the RPC was automatically selected from the internal limiting membrane (ILM) to the RNFL posterior boundary. Quantitative parameters of the macular retina include average vessel density of superficial (SVD) and deep (DVD) vascular complexes in the whole image (wSVD, %; wDVD, %) and the corresponding superior- and inferior-hemi values of SVD (sSVD, %; iSVD, %) and DVD (sDVD, %; iDVD, %). The slab of superficial retinal vascular complexes in OCT-A was automatically segmented from ILM to 10 *μ*m above the inner-plexiform layer (IPL), and the deep retinal vascular complexes were selected from 10 *μ*m above the IPL to 10 *μ*m below the outer plexiform layer (OPL). An automatic method provided by the commercial equipment were used for the segmentation of OCT and OCT-A images; otherwise, manual adjustment was performed to ensure that the scan was focused on the center of the disc/macular fovea and to correct inaccurate automated segmentation.

All statistical tests were performed using SPSS version 23.0 (https://www.ibm.com/analytics/spss-statistics-software). Categorized data are described as frequency and percentage, and quantitative data are presented as mean and standard deviation. The Shapiro–Wilk test was used to evaluate distribution. Student's *t*-test and one-way analysis of variance (ANOVA) were used in cases of normal distribution, while nonparametric statistical analyses (independent samples Mann–Whitney *U* or related-samples Wilcoxon test and Kruskal–Wallis test) were used for comparing variables between groups. The Pearson or Spearman method was used to analyze the correlations of the RPC or SVD with other parameters. A *p* value <0.05 was considered statistically significant.

## 3. Results

A total of 21 affected eyes and 19 unaffected eyes from 21 NAION patients and 2 fellow (unaffected) eyes were excluded from the analysis due to the existing optic atrophy. In this group, 3 cases had self-reported history of hypertension and one had diabetes. Twelve (57.14%) were male and 9 (42.86%) were female with a mean age of 54.67 ± 7.55 years. The average time after onset of symptoms at the initial visit was 5.7 ± 3.1 days. Repeated measurements of OCT and OCT-A parameters were performed in 21, 21, and 13 affected eyes with NAION at follow-up intervals of 1-2 weeks, 1-2 months, and 3–6 months, respectively. Eight affected eyes were lost to follow-up at 3–6 months. BCVA was worse in affected eyes (0.424 ± 0.326 LogMAR) than that of unaffected eyes (0.158 ± 0.155 LogMAR) at the initial visit (*p*=0.006). In affected eyes, a significant improvement of BCVA was found at the last follow-up visit (0.357 ± 0.326 LogMAR) compared to the initial assessment (*p*=0.021).

### 3.1. Comparison of OCT and OCT-A Parameters in ONH

**Figure 1:**
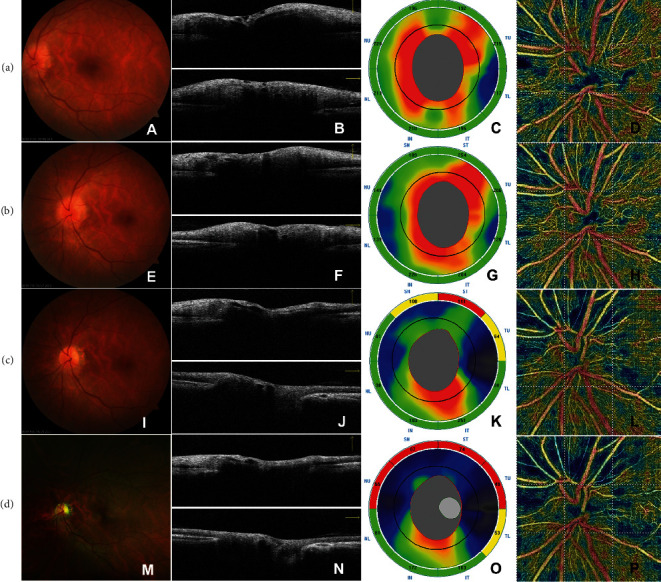
The time course of CFP, structural OCT, and Angio-OCT (OCT-A) findings in ONH in a case with NAION ((a) baseline: A∼D, (b) 1∼2 w: E∼H, (c) 1∼2 m: I∼L, and (d) 3∼6 m: M∼P). At presentation, CFP (A, E, I, M) indicated the occurrence of ODE at the initial visit (A), and the subsequent resolution (E, I) of ODE and the final pallor of optic disc (M). The B-scan OCT image of ONH (B, F, J, N; above, horizontal B-scan; bottom, vertical B-scan) showed that, at the initial diagnosis (B), there was edema of the optic disc and RNFL, which gradually subsided with the progress of the disease (F, J, N), and the RNFL around the upper optic disc became significantly thinner at 3–6 M (N). The RNFL thickness map (C, G, K, O) showed that the thickness of the RNFL at the initial visit (C) and 1∼2 w (G) follow-up significantly increased, which were followed by gradual decrease at 1∼2 m (K) and 3∼6 m (O) follow-ups, especially those in the superior sector, as the increasing areas of red or yellow color shown in the map. The vessel density map of Angio-Disc (D, H, L, P) showed an superiorly sectoral reduction of the vessel density of the RPC from the acute stage (D, H) to the late stage (L, P) of NAION, as the increasing areas of blue color shown in the map. CFP, color fundus photography. ONH, optical nerve head. NAION, nonarteritic anterior ischemic optic neuropathy. ODE, optic disc edema. RNFL, retinal nerve fiber layer. RPC, radial peripapillary capillary.

Compared with unaffected control eyes, wRNFL values in affected eyes were significantly higher at the initial visit (*p* ≤ 0.001) and the intervals of 1-2 weeks (*p* ≤ 0.001) and lower at 3–6 weeks after enrollment (*p* ≤ 0.001), while wRPC values were lower in affected eyes at all follow-up intervals (*p* ≤ 0.001 for all) (Table 1, Figure 1). Comparison within affected eyes of NAION: the overall differences of wRNFL and wRPC at the four intervals were statistically significant (*p* ≤ 0.001) (Table 1, Figure 1). Post hoc multiple comparisons showed a significant difference of wRPC values between intervals of baseline and 1-2/3–6 months and between 1-2 weeks and 3–6 months after enrollment (*p*=0.001, *p* ≤ 0.001, *p*=0.001), accompanied by significant differences between sRPC and iRPC observed at 1-2 months and 3–6 months follow-ups (*p*=0.016, *p*=0.013) (Table 1, Figure 1). Also, similar changes have been found in wRNFL values (Table 1, Figure 1).

### 3.2. Comparison of OCT and OCT-A Parameters in the Macular Retina

**Figure 2:**
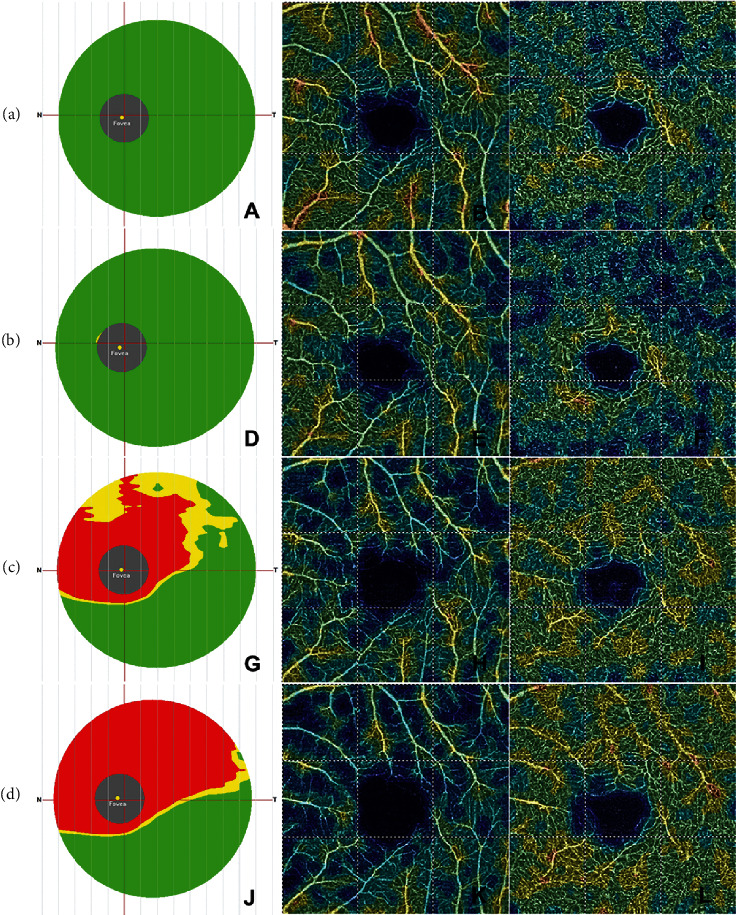
The time course of structural OCT and Angio-OCT findings in macular retina in the same patient in Figure 1 ((a) baseline, A∼C; (b) 1∼2 w, D∼F; (c) 1∼2 m, G∼I; (d) 3∼6 m, J∼L). The GCC thickness reference map (A, D, G, J) showed a significant decrease of the thickness of the GCC at 1∼2 m (G) and 3∼6 m (J) follow-ups compared to those at the initial visit (A) and 1∼2 w follow-up (D), as the increasing areas of red or yellow color shown in the map. The superficial vessel density map of Angio-Retina 3.0 mm (B, E, H, K) showed the reduction of SVD, which became sever with the time progress. The deep vessel density map of Angio-Retina 3.0 mm (C, F, I, L) showed that DVD decreased at the initial visit (C) and 1∼2 w follow-up (F), while there was slight increase of DVD at 1∼2 m (I) and 3∼6 m (L) follow-ups. GCC, ganglion cell complex; SVD, superficial vessel density; DVD, deep vessel density.

Compared with unaffected control eyes, wGCC and wSVD values in affected eyes were significantly lower at the follow-up intervals of 1-2 weeks, 1-2 months, and 3–6 months (*p*=0.002, ≤0.001, ≤0.001; *p*=0.008, ≤0.001, ≤0.001) (Table 2, Figure 2), while there was no significant difference of wDVD values between affected and unaffected eyes at all follow-up intervals (*p*=0.121, 0.044, 0.673, 0.600) (Table 2, Figure 2). Comparisons within affected eyes of NAION: the overall differences of wGCC and wSVD at four intervals were statistically significant (*p* ≤ 0.001 for both), whereas there were no significant differences for wDVD (*p* > 0.05) (Table 2, Figure 2). Post hoc multiple comparisons showed significant differences in wSVD values between the initial visit and 1-2 months or 3–6 months follow-ups and between 1-2 weeks and 3–6 months follow-ups (*p* ≤ 0.001, *p* ≤ 0.001, *p* ≤ 0.001), accompanied by significant differences between sSVD and iSVD observed at the 1-2 months follow-up (*p*=0.010) (Table 2, Figure 2). Also, similar changes have been found in wGCC values (Table 2, Figure 2).

Spearman's coefficients indicated significant relationships between wRPC and wRNFL at 3–6 months follow-ups (*r* = 0.626, *p*=0.022), between wSVD and wGCC at 1-2 weeks and 1-2 months follow-ups (*r* = 0.570, *r* = 0.436; *p*=0.007, *p*=0.048), and between wRNFL and wGCC at 1-2 months and 3–6 months follow-ups (*r* = 0.615, *r* = 0.621; *p*=0.003, *p*=0.024).

## 4. Discussion

Most cross-sectional studies to date addressing the atrophic stage (>3 months) of NAION have reported a consistent conclusion of significant reduction of peripapillary radial capillaries (RPCs) and [9, 12, 15] microvasculature in the macula by OCT-A [9, 16]. Also, these OCT-A-derived capillary density changes in RPC were spatially correlated with the severity of visual field defect and thinning of the peripapillary RNFL [9, 15]. Thus, it has been well accepted that there is an obligatory loss of capillary density resulting from the loss of corresponding layers of neural tissue in the pathological progression of NAION [9, 12, 15, 16]. A few studies concerning OCT-A in the acute stage of NAION show the defects in peripapillary microcirculation [13, 17, 18]. Song et al. [19] and Rebolleda et al. [13] have reported that there were no significant differences of reduced peripapillary vessel density in acute and chronic stages. However, they assessed the measurements with smaller scanning area [13] and compared the acute and chronic stages with the division of 21 days using two separate patient pools [19], which may be generally related to the comparison between 1-2 weeks and 1-2 months follow-up intervals in our cohort that correspond to the stage of resolution. In this cohort, in addition to the significant reduction of wRPC at enrollment compared to unaffected eyes, we also found that wRPC in NAION-affected eyes decreased significantly from the acute stage to the stages of resolution and atrophy, along with a more drastic reduction of sRPC, suggesting a sectoral reduction of RPC as the ODE subsided.

The most plausible explanation for an apparent decrease in RPC density during the acute phase could be the mechanical compression and impedance of the flow in the RPC from the edema of the optic nerve [13, 17], and the local attenuated signal strength caused by the edema of the tissue may also contribute a decrease in capillary density [17]. Additionally, it is possible that the reduced capillary density results from the progressive hypoxia and ischemia with the early axon loss which would be masked by the edema in some patients [1, 4].

Compared to peripapillary RNFL thinning, the pattern of GCL/GCC thinning may reflect earlier structural changes caused by ischemia of the optic nerve in NAION [20, 21]. Thinning of inner layers in the macula could be detected within one week after the ischemic event [20, 21] reflecting the early damage of the RGC and the axons [3, 20–23] and peak at 3–6 months and last for 6–12 months [3, 22, 23]. Our results showed that the corresponding wSVD in affected eyes was gradually decreased at 1–2 weeks follow-ups and aggravated at 1-2 and 3–6 months follow-ups along with worsening superior hemifield defect. At the stage of resolution, we found a significant correlation between the reduction of wSVD and wGCC, which has not been detected in previous reports. Together with the significant association between wGCC thinning and wRNFL, our results provide further data to support the hypothesis that thinning of GCL in the macula was secondary to the degeneration of peripapillary axons that are prone to suffer from hypoxia and ischemia [20, 23, 24]. There could be a vicious cycle about the neural-vascular interactions in the pathological progression of NAION [1, 24].

Although some patients showed mild reduction in wGCC and wSVD at the enrollment, the difference between affected and unaffected eyes was not significant enough to draw a confirmed conclusion, while, at the acute stage of NAION, the edema of RNFL in macula would mask the ability to detect significant thinning of the underlying layers of the GCL and IPL [23], and this may be part of the reasons for that the correlation between wSVD and wGCC particularly occurred at the stage of resolution. It might be that the OCT-A-derived capillary density in SVD shows decrease due to both the edema of the RNFL and/or early thinning of the underlying layers [23, 25]. As Augstburger et al. [9] proposed, the decrease of SVD in the 6 × 6 mm scan of OCT-A predominates near the large retinal vessels of the arcades and less at the parafoveal areas where the capillary network converges and forms an anastomotic ring [9, 24, 25]. Also, the axonal swelling or RNFL edema resulting from the blocked axoplasm and leading to impedance in flow of retinal vessels might be severer in the site closer to the optic disc. Fard et al. [26] have reported an early reduction of SVD and DVD in 6 × 6 mm scans at the acute stage within 2 weeks of onset. It might be that we missed the changes outside the 3 × 3 mm scanning area in the early stage. Also, it is possible that oral corticosteroid therapy accelerates the regression of ODE [14] and, therefore, makes the early reduction of SVD insignificant. Besides, an early reduction of wDVD in affected eyes has been detected in our study, which might be related to the presumption that the deep capillary vortexes [27] might be earlier or more frequent to compensate for the lower blood flow and hypoxia in the macula as previously reported in patients with hypertension and diabetes mellitus [28, 29].

Contrary to the previous results of decreased DVD in chronic stage with macular 6 × 6 mm OCT-A imaging [9, 16], our results with a 3 × 3 mm scan show that the wDVD did not decrease significantly with time procession. This could be explained by the penetration of coherent light and enhanced visibility of deep retinal vessel complexes resulted from severer thinning of inner layers, which needs further validation with longer observation. Although grading accuracy of OCT B-scan for 3 × 3 mm OCT-A was better than for 6 × 6 mm OCT-A which has limited vascular depth discrimination and is prone to induce overestimation of the measurements [25, 30], the limited 3 × 3 mm scanning area of OCT-A could miss those changes outside the confined area and, therefore, underestimate the results.

There are several limitations in our study, including its retrospective nature and the small number of subjects, and some patients were lost to follow-up at 3–6 months. Here, we focused on flow alterations with time, yet visual field loss of threshold was not analyzed in detail as in the previous studies [9, 13, 26]. Additionally, vasculature in prelaminar and laminar slabs was not evaluated due to the limits of the current technology, which may be more related to the ischemia mechanism of PCA.

## 5. Conclusions

This study was designed to analyze the microvascular flow impairment in the RPC and the macular retina from acute to atrophic stages in a cohort of patients with NAION. We provide further evidence to support that there is a sectorial reduction of vessel density in the RPC and macula with the disease progression of NAION from acute to atrophic stages, a classification associated with structural deficits.

## Figures and Tables

**Table 1:** OCT and OCT-A parameters in ONH in affected eyes of NAION.

Affected		Baseline (*n* = 21)	1∼2 w (*n* = 21)	1∼2 m (*n* = 21)	3∼6 m (*n* = 13)	*p* ^a^ value
RNFL (*μ*m) ($\bar{\chi }\pm s$)	wRNFL	179.24 ± 17.77	153.72 ± 25.19	111.90 ± 11.14	83.00 ± 14.35	≤0.001^*∗*^
	sRNFL	186.95 ± 23.18	156.29 ± 31.19	104.29 ± 15.73	74.23 ± 10.35	≤0.001^*∗*^
	iRNFL	171.67 ± 19.78	150.29 ± 26.53	119.52 ± 18.39	91.85 ± 22.28	≤0.001^*∗*^
	*p* ^si^ value	0.019^*∗*^	0.140	0.013^*∗*^	0.007^*∗*^	—

RPC (%) ($\bar{\chi }\pm s$)	wRPC	46.32 ± 2.63	44.29 ± 3.28	41.45 ± 3.43	38.22 ± 4.00	≤0.001^*∗*^
	sRPC	46.90 ± 3.69	44.29 ± 4.70	40.81 ± 4.13	36.53 ± 6.19	≤0.001^*∗*^
	iRPC	48.29 ± 5.55	46.75 ± 5.42	44.61 ± 5.55	42.75 ± 6.60	0.029^*∗*^
	*p* ^si^ value	0.266	0.035^*∗*^	0.016^*∗*^	0.013^*∗*^	—

Unaffected		Baseline (*n* = 19)	1∼2 w (*n* = 19)	1∼2 m (*n* = 19)	3∼6 m (*n* = 13)	*p* ^a^ value
	wRNFL	113.84 ± 13.16	113.42 ± 11.59	113.78 ± 11.74	116.38 ± 12.49	0.945
	wRPC	50.74 ± 2.00	51.04 ± 1.46	51.06 ± 1.58	50.76 ± 1.62	0.913
	*p* ^b^ value	≤0.001^*∗*^	≤0.001^*∗*^	0.856	≤0.001^*∗*^	—
	*p* ^c^ value	≤0.001^*∗*^	≤0.001^*∗*^	≤0.001^*∗*^	≤0.001^*∗*^	—

NAION, nonarteritic anterior ischemic optic neuropathy. ONH, optical nerve head. wRNFL (*μ*m), average thickness of the peripapillary retinal nerve fiber layer. wRPC (%), average capillary density of the radial peripapillary capillary. s/iRNFL (*μ*m), superior- and inferior-hemi thickness of the peripapillary retinal nerve fiber layer. s/iRPC (%), superior- and inferior-hemi capillary density of the radial peripapillary capillary. *p*^si^, *p* value for comparison of superior- and inferior-hemi values using the Wilcoxon test. *p*^a^, *p* value obtained using the nonparametric Kruskal–Wallis test for evaluating the overall differences among four intervals. *p*^b^/*p*^c^, *p* values for comparison of wRNFL/wRPC values in affected subjects versus unaffected eyes using the Wilcoxon test. ^*∗*^*p* value < 0.05 was considered statistically significant.

**Table 2:** OCT and OCT-A parameters in the macular retina in affected eyes of NAION.

Affected		Baseline (*n* = 21)	1∼2 w (*n* = 21)	1∼2 m (*n* = 21)	3∼6 m (*n* = 13)	*p* ^a^ value
GCC (*μ*m) ($\bar{\chi }\pm s$)	wGCC	103.67 ± 6.95	99.48 ± 7.69	89.24 ± 6.66	80.62 ± 8.62	≤0.001^*∗*^
	sGCC	102.67 ± 7.91	95.52 ± 8.80	84.62 ± 7.72	74.62 ± 9.46	≤0.001^*∗*^
	iGCC	104.62 ± 7.34	103.48 ± 8.80	93.85 ± 7.72	86.54 ± 10.37	≤0.001^*∗*^
	*p* ^si^ value	0.114	0.001^*∗*^	≤0.001^*∗*^	0.002^*∗*^	—

SVD (%) ($\bar{\chi }\pm s$)	wSVD	46.07 ± 2.09	43.70 ± 2.39	41.10 ± 2.63	37.16 ± 3.77	≤0.001^*∗*^
	sSVD	46.19 ± 2.16	43.91 ± 2.43	40.32 ± 3.12	36.92 ± 3.89	≤0.001^*∗*^
	iSVD	45.89 ± 2.30	43.63 ± 2.73	41.75 ± 2.47	36.88 ± 4.06	≤0.001^*∗*^
	wDVD	45.82 ± 2.96	45.37 ± 3.68	46.99 ± 2.39	47.44 ± 3.35	0.075
	*p* ^si^ value	0.149	0.626	0.010^*∗*^	0.807	—
Unaffected		(*n* = 19)	(*n* = 19)	(*n* = 19)	(*n* = 13)	*p* ^a^ value
	wGCC	106.68 ± 7.61	107.58 ± 6.63	106.79 ± 7.22	106.92 ± 7.51	0.971
	wSVD	45.49 ± 1.97	45.52 ± 1.87	45.44 ± 1.89	45.41 ± 1.67	0.999
	wDVD	47.60 ± 3.20	47.64 ± 2.89	47.67 ± 3.02	46.94 ± 2.66	0.876
	*p* ^b^ value	0.304	0.002^*∗*^	≤0.001^*∗*^	≤0.001^*∗*^	—
	*P* ^c^ value	0.444	0.008^*∗*^	≤0.001^*∗*^	≤0.001^*∗*^	—
	*P* ^d^ value	0.121	0.044^*∗*^	0.673	0.600	—

NAION, nonarteritic anterior ischemic optic neuropathy. wGCC (*μ*m), average ganglion cell complex thickness. wSVD (%), average superficial vessel density. wDVD (%), average deep vessel density. s/iGCC (*μ*m), superior- and inferior-hemi thickness of macular ganglion cell complex. s/iSVD (%), s/iDVD(%), superior- and inferior-hemi values of superficial and deep vessel density. *p*^si^, *p* value for comparison of superior- and inferior-hemi values using the Wilcoxon test. *p*^a^, *p* value obtained using the nonparametric Kruskal–Wallis test for evaluating the overall differences among four intervals. *p*^b^/*p*^c^/*p*^d^, *p* values for comparison of wGCC/wSVD/wDVD values in affected subjects versus unaffected eyes using the Wilcoxon test. ^*∗*^*p* value<0.05 was considered statistically significant.

## Data Availability

The datasets used and/or analyzed during the current study are available from the corresponding author on reasonable request.
